# Docosahexaenoic acid antagonizes the boosting effect of palmitic acid on LPS inflammatory signaling by inhibiting gene transcription and ceramide synthesis

**DOI:** 10.1371/journal.pone.0193343

**Published:** 2018-02-23

**Authors:** Junfei Jin, Zhongyang Lu, Yanchun Li, L. Ashley Cowart, Maria F. Lopes-Virella, Yan Huang

**Affiliations:** 1 Division of Endocrinology, Diabetes and Medical Genetics, Department of Medicine, Medical University of South Carolina, Charleston, United States of America; 2 Ralph H. Johnson Veterans Affairs Medical Center, Charleston, United States of America; 3 Department of Biochemistry and Molecular Biology, Medical University of South Carolina, Charleston, United States of America; University of Illinois, UNITED STATES

## Abstract

It is well known that saturated fatty acids (SFAs) and unsaturated fatty acid, in particular omega-3 polyunsaturated fatty acids (n-3 PUFAs), have different effects on inflammatory signaling: SFAs are pro-inflammatory but n-3 PUFAs have strong anti-inflammatory properties. We have reported that palmitic acid (PA), a saturated fatty acid, robustly amplifies lipopolysaccharide (LPS) signaling to upregulate proinflammatory gene expression in macrophages. We also reported that the increased production of ceramide (CER) via sphingomyelin (SM) hydrolysis and CER de novo synthesis plays a key role in the synergistic effect of LPS and PA on proinflammatory gene expression. However, it remains unclear if n-3 PUFAs are capable of antagonizing the synergistic effect of LPS and PA on gene expression and CER production. In this study, we employed the above macrophage culture system and lipidomical analysis to assess the effect of n-3 PUFAs on proinflammatory gene expression and CER production stimulated by LPS and PA. Results showed that DHA strongly inhibited the synergistic effect of LPS and PA on proinflammatory gene expression by targeting nuclear factor kappa B (NFκB)-dependent gene transcription. Results also showed that DHA inhibited the cooperative effect of LPS and PA on CER production by targeting CER de novo synthesis, but not SM hydrolysis. Furthermore, results showed that myriocin, a specific inhibitor of serine palmitoyltransferase, strongly inhibited both LPS-PA-stimulated CER synthesis and proinflammatory gene expression, indicating that CER synthesis is associated with proinflammatory gene expression and that inhibition of CER synthesis contributes to DHA-inhibited proinflammatory gene expression. Taken together, this study demonstrates that DHA antagonizes the boosting effect of PA on LPS signaling on proinflammatory gene expression by targeting both NFκB-dependent transcription and CER de novo synthesis in macrophages.

## Introduction

Metabolic endotoxemia is a pathological condition in which circulating lipopolysaccharide (LPS) is elevated in patients with obesity or diabetes as a result of high-fat diet (HFD)-increased intestinal permeability that facilitates the translocation of microbiome-derived LPS from the intestine to the bloodstream [1, 2]. It has been shown that bacterial LPS activity in human serum is associated with dyslipidemia, obesity and chronic inflammation [3]. In addition to LPS, plasma free saturated fatty acid (SFA) is also increased in patients with obesity or diabetes and promotes inflammation and cardiovascular disease [4–6]. When both LPS and SFA in circulation are elevated, they may act in concert to boost a strong proinflammatory response that cannot be generated by LPS or SFA alone. Indeed, our recent study supported the above notion as it showed a cooperative stimulation of atherogenesis by combining treatment of LPS with feeding SFA-rich HFD in animal models [7]. Our *in vitro* study also showed that palmitic acid (PA), the most abundant SFA in plasma [8], amplified LPS-triggered signaling for upregulating proinflammatory genes such as interleukin 6 (IL-6) in macrophages [9]. Furthermore, our study to explore the underlying mechanisms showed that the stimulation of ceramide (CER) production by PA and LPS plays a key role in the synergistic effect of PA and LPS on proinflammatory gene expression [9].

CER is mainly generated by the breakdown of sphingomyelin by sphingomyelinases (SMases) that include acid SMase (aSMase), neutral SMase (nSMase) or alkaline SMase [10]. Of these SMases, aSMase and nSMase are considered as the major SMases for the production of cellular CER. CER can be also generated by de novo synthesis catalyzed by enzymes such as serine palmitoyl transferase and CER synthase. CER plays key roles in a variety of cellular responses, regulating cell growth, differentiation, senescence, apoptosis and inflammation [11].

In contrast to the proinflammatory effects of SFAs, omega-3 (n-3) polyunsaturated fatty acids (PUFAs) have anti-inflammatory properties [12, 13]. A recent clinical study showed that supplementation with n-3 PUFAs in obese patients reduced proinflammatory cytokines and C-reactive protein, and increased adiponectin in circulation [14]. Another clinical study also showed that n-3 PUFAs reduced the circulating level and adipose tissue expression of proinflammatory cytokines in obese patients [15]. Although the beneficial effects of n-3 PUFAs on obesity or diabetes have been increasingly appreciated in recent years [16, 17], the underlying mechanisms whereby n-3 PUFAs inhibit inflammation have not been fully understood.

In this study, we have employed the cell model in which PA amplifies LPS signaling for proinflammatory molecule expression [9] to determine if n-3 PUFAs is capable of antagonizing PA-boosted LPS proinflammatory signaling. Moreover, since we have shown in this cell model that increased production of CER, a bioactive sphingolipid [18], is involved in the synergistic stimulation of proinflammatory cytokine expression by LPS and PA [9], we also determined if n-3 PUFAs antagonizes the stimulatory effect of LPS and PA on CER production.

## Materials and methods

### Cell culture

RAW264.7 cells, the murine macrophages used extensively in the investigations of the role of macrophages in inflammation [19], were purchased from the American Type Culture Collection (ATCC, Manassas, VA) and grown in Dulbecco's modified Eagle's medium (DMEM) (ATCC, Manassas, VA) supplemented with 10% heat-inactivated fetal bovine serum (FBS) (HyClone, Logan, UT). The cells were maintained in a 37°C, 90% relative humidity, 5% CO_2_ environment. The cells were seeded into 12-well plates at a density of 2 x 10^5^/well and allowed to grow for 24 h before treatment. The cells at passage 5–8 were used and were 80% confluent at the treatment. For cell treatment, LPS from E. *coli* serotype 055:B5 (Sigma, St. Louis, MO) was used. The LPS was highly purified by phenol extraction and gel filtration chromatography, and was cell culture tested.

### Fatty acid preparation

The PA, DHA, eicosapentaenoic acid (EPA), oleic acid (OA), linoleic acid (LA) and palmitoleic acid (POA) used in this study were purchased from Sigma (St. Louis, MO) and prepared as described previously [20, 21]. Briefly, fatty acids were dissolved in 0.1 N NaOH and 70% ethanol at 70°C to make concentration of 50 mM. The solution was kept at 55°C for 10 min, mixed, and brought to room temperature before treatment.

### Enzyme-linked immunosorbent assay (ELISA)

IL-6 in medium was quantified using sandwich ELISA kits according to the protocol provided by the manufacturer (Biolegend, San Diego, CA).

### Real-time polymerase chain reaction (PCR)

Total RNA was isolated from cells using RNeasy minikit (Qiagen, Santa Clarita, CA). First-strand complementary DNA (cDNA) was synthesized with the iScript^TM^ cDNA synthesis kit (Bio-Rad Laboratories, Hercules, CA) using 20 μl of reaction mixture containing 0.5 μg of total RNA, 4 μl of 5x iScript reaction mixture, and 1 μl of iScript reverse transcriptase. The complete reaction was cycled for 5 minutes at 25 ^o^C, 30 minutes at 42 ^o^C and 5 minutes at 85°C using a PTC-200 DNA Engine (MJ Research, Waltham, MA). The reverse transcription reaction mixture was then diluted 1:10 with nuclease-free water and used for PCR amplification in the presence of the primers [mouse serine palmitoyltransferase (SPT)1: 5’ primer sequence, AGTGGTGGGAGAGTC CCTTT; 3’ primer sequence, CAGTGACCACAACCCTGATG]. Primers were synthesized by Integrated DNA Technologies, Inc. (Coralville, IA). Real-time PCR was performed in duplicate using 25 μl of reaction mixture containing 1.0 μl of RT mixture, 0.2 μM of both primers, and 12.5 μl of iQ^TM^ SYBR Green Supermix (Bio-Rad Laboratories). Real-time PCR was run in the iCycler^TM^ real-time detection system (Bio-Rad Laboratories) with a two-step method. The hot-start enzyme was activated (95°C for 3 min) and cDNA was then amplified for 40 cycles consisting of denaturation at 95°C for 10 sec and annealing/extension at 60°C for 45 sec. A melt-curve assay was then performed (55°C for 1 min and then temperature was increased by 0.5°C every 10 sec) to detect the formation of primer-derived trimmers and dimmers. Mouse glyceraldehyde 3-phosphate dehydrogenase (GAPDH) was used as a control (5’ primer sequence, GDDTTCCGTGTTCCTACC; 3’ primer sequence, GCCTGCTTCACCACCTTC). Data were analyzed with the iCycler iQ^TM^ software. The average starting quantity (SQ) of fluorescence units was used for analysis. Quantification was calculated using the SQ of targeted cDNA relative to that of GAPDH cDNA in the same sample.

### PCR arrays

RNA isolated from duplicate samples was pooled for the reverse transcription to make cDNA. The first-strand cDNA was synthesized from RNA using RT^2^ First Strand Kit (SuperArray Bioscience Corp., Frederick, MD). Mouse Toll-like receptor (TLR) pathway-focused PCR Arrays (SuperArray Bioscience Corp.) were performed using 2X SuperArray RT^2^ qPCR master mix and the first strand cDNA by following the instruction from the manufacturer.

### Transfection and luciferase activity assay

To study IL-6 transcription, RAW 264.7 cells were transiently transfected with p1168huIL6P-luc+ plasmids (Belgian Co-ordinated Collections of Micro-organisms, BCCM^TM^, Brussels, Belgium) (1 μg/10^6^ cells) with transfection reagent FuGENE HD (Promega Corporation, Madison, WI). Cells were cotransfected with the *Renilla* luciferase reporter plasmid pRL-TK (Promega) (50 ng/10^6^ cells) as an internal control. Twenty-four hours after transfection, the cells were stimulated with LPS, PA or both LPS and PA. To study nuclear factor kappa B (NFκB)-mediated transcription, RAW264.7 cells were transfected with 1 μg of NFκB Cignal Reporter (Qiagen Inc., Valencia, CA) using FuGENE^®^ HD as transfection reagent for 24 h. The renilla luciferase constructs were used as control. The cells were then treated with fresh medium containing 1 ng/ml of LPS, 100 μM of PA or LPS plus PA for 12 or 24 h. After the treatment, the cells were rinsed with cold PBS and lysed with the buffer from Dual-Luciferase Reporter Assay System (Promega). Both firefly and renilla luciferase levels were assayed in a luminometer using the dual-luciferase reporter assay reagents (Promega) according to the instruction from the manufacturer. The firefly luciferase levels were normalized to the renilla luciferase levels.

### Lipidomics

RAW264.7 cells were collected, fortified with internal standards, extracted with ethyl acetate/isopropyl alcohol/water (60:30:10, v/v/v), evaporated to dryness, reconstituted in 100 μl of methanol and subjected to lipidomic analysis of CER and SM as described previously [22]. Simultaneous ESI/MS/MS analyses of CERs and SMs were performed on a Thermo Finnigan TSQ 7000 triple quadrupole mass spectrometer operating in a multiple reaction monitoring positive ionization mode. The phosphate contents of the lipid extracts were used to normalize the MS measurements of sphingolipids and measured with a standard curve analysis and a colorimetric assay of ashed phosphate [23].

### aSMase activity assay

RAW264.7 cells were treated and cell lysate was then used for aSMase activity assay using an aSMase assay kit (Cayman Chemical, Ann Arbor, Michigan) according to the manufacture’s instructions.

### Statistical analysis

The experiments were performed in duplicates for 3 times and the data were presented as Least Squares means ± SD. One-way ANOVA was performed to determine the statistical significance among different experimental groups. A value of *P*<0.05 was considered significant.

## Results

### DHA strongly inhibits the upregulation of proinflammatory genes by LPS or LPS plus PA

**Fig 1:**
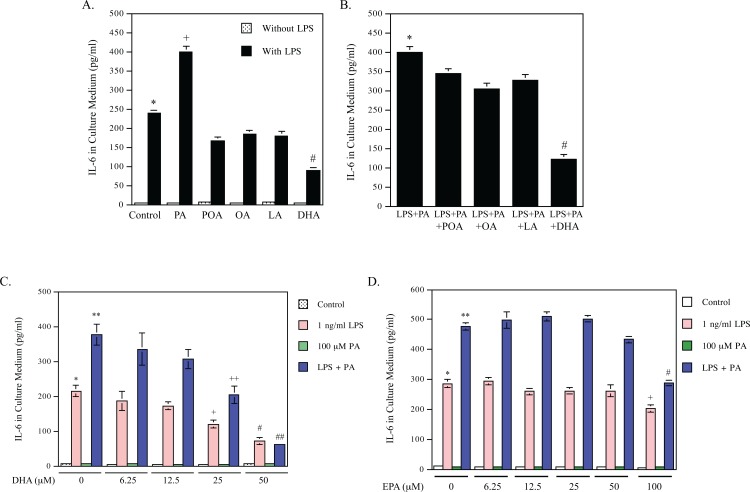
DHA inhibits IL-6 expression stimulated by LPS or LPS plus PA. A. RAW264.7 macrophages were treated with 100 μM of palmitic acid (PA), palmitoleic acid (POA), oleic acid (OA), linoleic acid (LA), or docosahexaenoic acid (DHA) in the absence or presence of 1 ng/ml of LPS for 24 h. After treatment, IL-6 in culture medium was quantified using ELISA. + vs. #, *p*<0.01; # vs. *, *p*<0.01. B. RAW264.7 macrophages were treated with both 1 ng/ml LPS and 100 μM of PA in the absence or presence of 100 μM of POA, OA, LA or DHA for 24 h. After treatment, IL-6 in culture medium was quantified using ELISA. # vs. *, *p*<0.01. C and D. RAW264.7 macrophages were treated with 1 ng/ml of LPS, 100 μM of PA or both 1 ng/ml LPS and 100 μM of PA in the absence or presence of different concentrations of DHA (C) or eicosapentaenoic acid (EPA) (D) for 24 h. After treatment, IL-6 in culture medium was quantified using ELISA. In panel C: ** vs. *, *p*<0.01; + vs. *, *p*<0.05; # vs. *, *p*<0.01; ++ vs. **, *p*<0.05; ## vs. **, *p*<0.01. In panel D: ** vs. *, *p*<0.01; + vs. *, *p*<0.05; # vs. **, *p*<0.01.

To compare the effects of different free fatty acids including SFA, monounsaturated fatty acid (MUFA) and PUFA on LPS-induced secretion of IL-6, a major proinflammatory cytokine [24], we used PA as a SFA, OA and POA as MUPAs, LA as a n-6 PUFA, DHA and EPA as n-3 PUFAs in this study. As shown in Fig 1A, while none of these fatty acids stimulated IL-6 secretion by itself, PA is the only fatty acid to have a synergy with LPS to stimulate IL-6 secretion. In contrast to PA, DHA inhibited LPS-induced IL-6 secretion by 62% and POA, OA and LA inhibited LPS-induced IL-6 secretion by 30%, 23%, 25%, respectively. Next, we determined the effect POA, OA, LA or DHA on the synergistic effect of LPS and PA on IL-6 secretion. As shown in Fig 1B, while POA, OA, LA reduced LPS-PA-stimulated IL-6 secretion by 14%, 24% and 19%, respectively, DHA markedly attenuated it by 70%. Our study further showed that DHA inhibited IL-6 secretion stimulated by LPS or LPS plus PA in a dose-dependent manner and DHA at 50 μM inhibited IL-6 secretion stimulated by LPS or LPS plus PA by 70% and 80%, respectively (Fig 1C). EPA, another ɷ-3 PUFA, was less potent than DHA in the inhibition of IL-6 secretion stimulated by LPS or LPS plus PA (Fig 1D).

To further elucidate the anti-inflammatory effect of DHA on the gene expression in macrophages in response to LPS or LPS plus PA, we profiled the gene expression using a Toll-like receptor (TLR) pathway-based PCR array. Data (Table 1) showed the synergistic effect of LPS and PA on the expression of a number of genes including monocyte chemoattractant protein 1 (MCP-1), CD86, colony stimulating factor 3 (CSF3), IL-1α, IL-1β, IL-6 and cyclooxygenase-2 (COX-2). For example, while LPS and PA alone induced MCP-1 expression by 18- and 11-fold, respectively, the combination of LPS and PA increased MCP-1 expression by 48-fold. Strikingly, although DHA did not inhibit the baseline expression of these genes, it potently antagonized the combined stimulation by LPS and PA of the expression of the above proinflammatory genes by 70–98%. It is noteworthy that the percent inhibition by DHA on the combined effect of LPS and PA appears to be higher than that on the effect of LPS or PA alone (Table 1). Interestingly, DHA exerted a stimulatory effect on the expression of IL-10, an anti-inflammatory cytokine [25]. While LPS, PA and LPS plus PA increased IL-10 expression by 2-, 2- and 4-fold, respectively, DHA further augmented the increases to 7-, 7- and 14-fold.

**Table 1:** The effect of DHA on gene expression stimulated by LPS, PA or LPS plus PA.

Genes	Fold increase by:			% inhibition by DHA	Fold increase by:		% inhibition by HDA	Fold increase by:		% inhibition by HDA
	DHA	LPS	LPS+DHA		PA	PA+DHA		LPS+PA	LPS+PA+DHA	
MCP-1	11	18	11	36	11	10	9	48	6	87
CD86	2	5	2	61	6	3	55	10	3	70
CSF3	3	3	4	-	7	2	73	95	3	97
IL-1α	3	10	1	87	6	2	74	30	5	84
IL-1β	1	10	3	71	3	4	-	29	4	87
IL-6	3	39	2	96	10	2	77	196	3	98
COX-2	1	3	2	33	5	2	67	11	2	81
IL-10	5	2	7	(up 288%)	2	7	(up 337%)	4	14	(up 339%)

RAW264.7 cells were treated with 1 ng/ml of LPS, 100 μM of palmitic acid (PA) or LPS plus PA in the absence or presence of 100 μM of DHA for 24 h and RNA was isolated from duplicate wells, combined and subjected to gene expression analysis using a Toll-like receptor (TLR) pathway-focused PCR array as described in the Methods. Full names for the abbreviations of the genes: MCP-1, monocyte chemoattractant protein 1; CSF3, colony stimulating factor 3; COX-2, cyclooxygenase-2.

### DHA and other unsaturated fatty acids inhibit the effect of LPS and PA on CER synthesis

**Fig 2:**
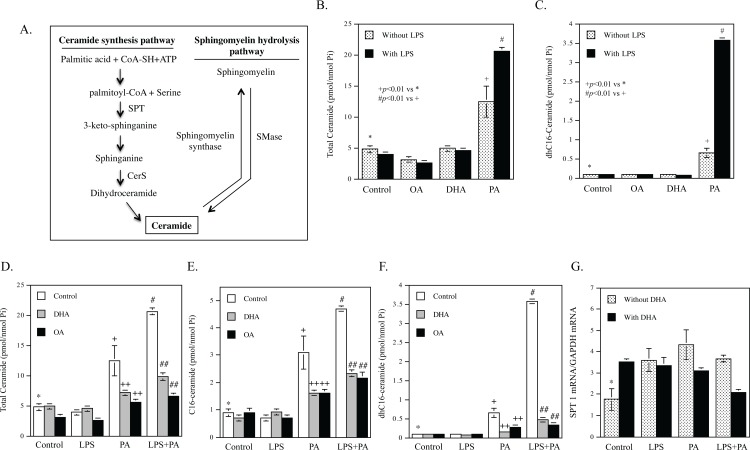
DHA inhibits the CER production increased by LPS and PA. A. Diagram for CER de novo synthesis and sphingomyelin hydrolysis pathways. SPT, serine palmitoyltransferase; CerS, ceramide synthase; SMase, sphingomyelinase. B and C. LPS and PA synergistically stimulate total (B) and dhC16-CER production (C). RAW264.7 macrophages were treated with 100 μM of OA, DHA or PA in the absence or presence of 1 ng/ml of LPS for 12 h. After treatment, total and dhC16-CER were quantified using lipidomics. + vs. *, *p*<0.01; # vs. +, *p*<0.01. D-F. Either DHA or OA inhibited PA- or LPS plus PA-stimulated CER production. RAW264.7 macrophages were treated with 1 ng/ml LPS, 100 μM PA or both LPS and PA in the absence or presence of 100 μM DHA or OA for 12 h. After treatment, total CER (D), C16-CER (E) and dhC16-CER (F) were quantified using lipidomics. + vs. *, *p*<0.01; # vs. +, *p*<0.01; ++ vs. +, *p*<0.01; ## vs. #, *p*<0.01. G. DHA inhibits SPT1 mRNA expression stimulated by LPS and PA. RAW264.7 macrophages were treated with 1 ng/ml LPS, 100 μM PA or both LPS and PA in the absence or presence of 100 μM of DHA for 12 h. After treatment, SPT1 mRNA was quantified using real-time PCR. * vs. +, *p*<0.05; * vs. #, *p*<0.05; * vs. ^, *p*<0.05; ^^ vs. ^, *p*<0.01.

CER is generated by either CER synthesis or SM hydrolysis [26] (Fig 2A). In addition, it is also generated by reacylation of sphingosine through the salvage pathway [27]. We have shown in our previous study that LPS and PA synergistically increase CER generation by stimulating both CER de novo synthesis and SM hydrolysis, and the increased CER in turn plays an important role in the upregulation of proinflammatory molecules [9]. In this study, we examined the effect of DHA on the stimulation of CER generation by LPS and PA. To determine if the effect is DHA specific, we compared the effect of DHA with that of OA.

Data from lipidomic analysis showed that DHA had no effect on total CER, OA reduced total CER by 35%, but PA increased total CER by 2.59-fold (Fig 2B and Table 2). Furthermore, LPS had no effect on total CER in control cells and cells treated with OA or DHA, but augmented PA-increased total CER by 44% (Fig 2B and Table 2). Similarly, PA increased dihydro (dh)C16-CER and LPS further augmented PA-increased dhC16-CER production by 5.5-fold (Fig 2C and Table 2). DHA had no effect on the baseline level of CER, but significantly inhibited the stimulatory effect of PA and LPS plus PA on total CER content by 46% and 52%, respectively (Fig 2D and Table 2). Interestingly, OA also exerted inhibition on total CER content increased by PA and PA plus LPS by 55% and 68%, respectively (Fig 2D). DHA or OA also inhibited C16-CER (Fig 2E) and dhC16-CER generation (Fig 2F) stimulated by PA or PA plus LPS. Given that dhC16-CER is an intermediate during CER de novo synthesis [28], these results indicate that DHA or OA inhibits PA- or LPS plus PA-stimulated CER de novo synthesis. In addition to dhC16-CER and C16-CER, DHA or OA also attenuated the stimulation of C18-, C20-, C22-CER production by PA or LPS plus PA (Table 2). Results from our lipidomic analysis also showed that POA and LA had similar inhibitory effect as DHA and OA on CER de novo synthesis (data not shown).

**Table 2:** The effect of DHA on CER content regulated by LPS, PA or LPS plus PA.

		dhC16-CER	C16-CER	C18-CER	C20-CER	C22-CER	C24-CER	C24:1-CER	Total CER
Control	Control	0.10±0.01 ^g1^	0.91±0.13 ^g2^	0.10±0.02	0.11±0.01	0.51±0.05	1.61±0.17	1.31±0.14	4.80±0.56 ^g^
	LPS	0.12±0.01	0.72±0.05	0.11±0.01	0.11±0.01	0.42±0.02	1.52±0.08	1.10±0.06	3.90±0.26
	PA	0.65±0.12 ^a1^	3.09±0.60 ^a2^	0.82±0.19	0.47±0.14	2.86±0.60	2.73±0.49	1.51±0.32	12.43±2.53 ^a^
	LPS+PA	3.58±0.06 ^b1^	4.70±0.08 ^b2^	1.60±0.22	0.88±0.01	4.29±0.09	3.23±0.01	1.87±0.04	20.58±0.56 ^b^
DHA	Control	0.10±0.01	1.10±0.02	0.11±0.01	0.10±0.01	0.52±0.04	1.60±0.03	1.40±0.05	4.90±0.16
	LPS	0.08±0.01	0.91±0.01	0.10±0.01	0.04±0.01	0.40±0.02	1.52±0.01	1.30±0.03	4.52±0.10
	PA	0.16±0.01 ^c1^	1.62±0.08 ^c2^	0.22±0.02	0.15±0.03	1.07±0.04	2.30±0.18	1.39±0.09	7.13±0.47 ^c^
	LPS+PA	0.47±0.02 ^d1^	2.33±0.12 ^d2^	0.42±0.01	0.26±0.03	1.64±0.17	2.81±0.06	1.65±0.23	9.81±0.66 ^d^
OA	Control	0.10±0.01	0.90±0.15	0.10±0.01	0.10±0.01	0.2±0.04	0.30±0.04	1.30±0.11	3.10±0.43
	LPS	0.10±0.01	0.70±0.03	0.10±0.01	0.1±0.01	0.2±0.01	0.3±0.02	0.9±0.09	2.60±0.18
	PA	0.27±0.01 ^e1^	1.62±0.12 ^e2^	0.39±0.02	0.14±0.03	0.67±0.02	0.72±0.07	1.59±0.06	5.61±0.34 ^e^
	LPS+PA	0.33±0.02 ^f1^	2.15±0.23 ^f2^	0.43±0.06	0.15±0.01	0.74±0.04	0.83±0.05	1.74±0.11	6.59±0.51 ^f^

a, *p*<0.01 vs g

b, *p*<0.01 vs a and g

c, *p*<0.01 vs a

d, *p*<0.01 vs b

e, p<0.01 vs a

f, p<0.01 vs b.

a1, *p*<0.01 vs g1

b1, *p*<0.01 vs a1 and g1

c1, *p*<0.01 vs a1

d1, *p*<0.01 vs b1

e1, *p*<0.01 vs a1

f1, *p*<0.01 vs b1.

a2, *p*<0.01 vs g2

b2, *p*<0.01 vs a2 and g2

c2, *p*<0.01 vs a2

d2, *p*<0.01 vs b2

e2, *p*<0.01 vs a2

f2, *p*<0.01 vs b2.

The concentration unit of the values in the table: pmole/nmol phosphate.

Since it has been shown that LPS upregulated the mRNA expression of SPT, a rate-limiting enzyme in CER de novo synthesis [29–31], we tested our hypothesis that DHA inhibits SPT1 mRNA expression. Results (Fig 2G) showed that LPS, PA or LPS plus PA increased SPT1 mRNA expression in the absence of DHA. Interestingly, DHA significantly attenuated LPS plus PA-stimulated SPT mRNA expression.

### DHA has no effect on the synergistic stimulation of SM hydrolysis by LPS and PA

**Fig 3:**
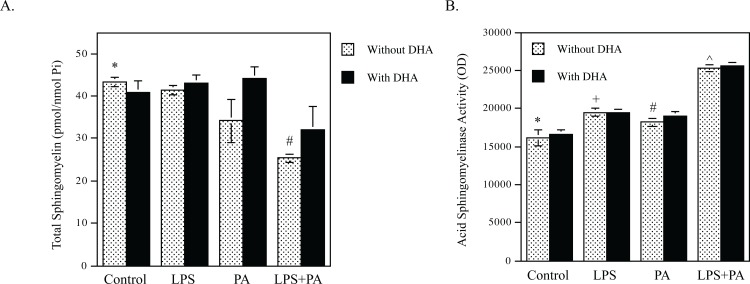
DHA has no effect on SM hydrolysis and ASMase activity stimulated by LPS and PA. A. RAW264.7 macrophages were treated with 1 ng/ml of LPS, 100 μM of PA or both 1 ng/ml LPS and 100 μM of PA in the absence or presence of 100 μM of DHA for 12 h. After treatment, cellular sphingomyelin was quantified using lipidomics. * vs. #, *p*<0.01. B. RAW264.7 macrophages were treated with 1 ng/ml of LPS, 100 μM of PA or both 1 ng/ml LPS and 100 μM of PA in the absence or presence of 100 μM of DHA for 2 h. After treatment, cellular ASMase activity was determined as described in the Methods. * vs. +, *p*<0.05; * vs. #, *p*<0.05; * vs. ^, *p*<0.05.

Since we have shown that LPS and PA synergistically stimulate CER production by increasing not only CER de novo synthesis, but also SM hydrolysis [9], we determined the effect of DHA on LPS-PA-stimulated SM hydrolysis. Data showed that while LPS and PA synergistically reduced SM content, DHA did not change SM content significantly (Fig 3A and Table 3). Furthermore, since we have shown that aSMase is responsible for increased SM hydrolysis in response to LPS and PA [9], we determined the effect of DHA on aSMase activity stimulated by LPS and PA. Results showed that while LPS and PA cooperatively stimulated aSMase activity, DHA did not attenuate LPS-PA-stimulated aSMase activity (Fig 3B).

**Table 3:** The effect of DHA on sphingomyelin content regulated by LPS, PA or LPS plus PA.

		C14-SM	C16-SM	C18-SM	C20-SM	C22-SM	C22:1-SM	C24-SM	C24:1-SM	Total SM
Without DHA	Control	0.86±0.21	24.87±4.87^a1^	2.18±0.87	0.66±0.19	3.76±0.73	0.70±0.18	3.00±1.09 ^a2^	6.98±2.13 ^a3^	43.30±0.20 ^a^
	LPS	0.95±0.18	24.5±4.79	1.84±0.79	0.59±0.15	3.35±0.71	0.68±0.12	3.08±0.97	5.80±2.08	41.40±0.06
	PA	0.59±0.01	20.56±1.51	2.36±1.08	0.66±0.20	2.98±1.05	0.60±0.11	1.73±1.09	4.44±1.95	34.16±7.08
	LPS+PA	0.46±0.05	16.81±2.23^b1^	1.52±0.48	0.38±0.09	1.75±0.41	0.37±0.07	1.01±0.54 ^b2^	2.85±1.09 ^b3^	25.31±0.44 ^b^
With DHA	Control	1.05±0.15	25.15±0.69	1.95±0.28	0.58±0.06	3.24±0.49	0.67±0.12	2.25±0.71	5.60±2.03	40.80±2.91
	LPS	1.22±0.26	28.49±3.79	1.78±0.14	0.53±0.01	2.83±0.19	0.63±0.06	2.22±0.29	4.89±1.37	42.91±1.96
	PA	0.75±0.26	28.00±3.26	2.44±0.05	0.72±0.06	4.01±0.51	0.57±0.01	2.57±0.08	4.80±1.18	44.10±2.80
	LPS+PA	0.67±0.31	21.34±5.04	1.73±0.35	0.45±0.09	2.66±0.74	0.43±0.08	1.42±0.01	3.25±0.30	32.16±6.38

b, *p*<0.01 vs a

b1, *p*<0.01 vs a1

b2, *p*<0.01 vs a2

b3, *p*<0.01 vs a3.

The concentration unit of the values in the table: pmole/nmol phosphate.

### DHA inhibits NFκB-mediated transcriptional activation by LPS and PA

**Fig 4:**
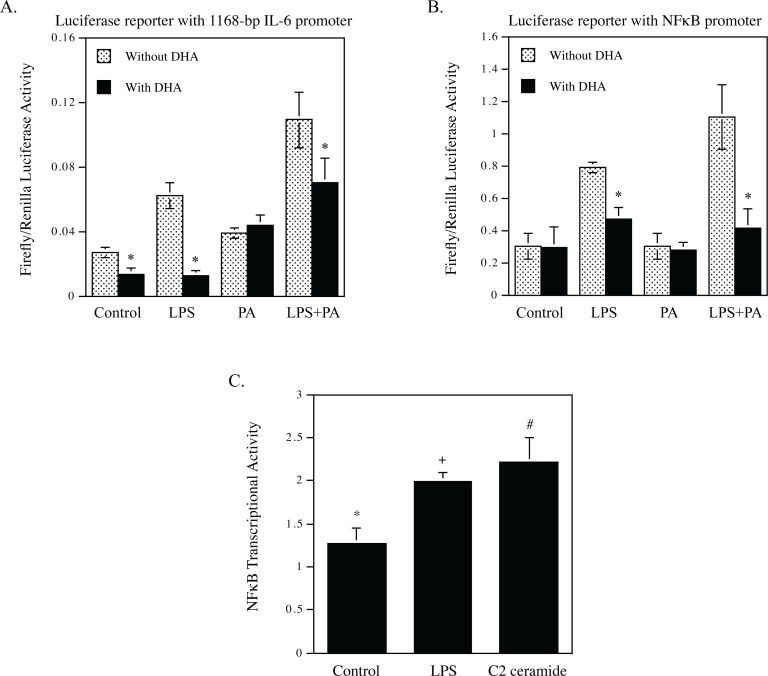
DHA inhibits IL-6 transcription and NFκB transcriptional activity. A. RAW264.7 macrophages were transfected with plasmids containing an 1168-bp IL-6 promoter sequence and luciferase reporter gene for 24 h. After the transfection, the macrophages were treated with 1 ng/ml of LPS, 100 μM of PA or both LPS and PA for 24 h and cellular firefly and renilla luciferase activities were assayed. The ratios of firefly vs. renilla luciferase were calculated. * vs. cells with the same treatment in the absence of DHA, *p*<0.01. B. RAW264.7 macrophages were transfected with DNA construct containing the tandem repeats of NFκB transcriptional response element in the promoter and luciferase reporter gene for 24 h. After the transfection, the macrophages were treated with 1 ng/ml of LPS, 100 μM of PA or both LPS and PA for 24 h and cellular firefly and renilla luciferase activities were assayed. The ratios of firefly vs. renilla luciferase were calculated. The ratios of firefly vs. renilla luciferase activity were calculated. * vs. cells with the same treatment in the absence of DHA, *p*<0.01. C. RAW264.7 macrophages were transfected with DNA construct containing the tandem repeats of NFκB transcriptional response element in the promoter and luciferase reporter gene for 24 h. After the transfection, the macrophages were treated with 1 ng/ml of LPS or 50 μM of C2-CER for 24 h and cellular firefly and renilla luciferase activities were then assayed. The ratios of firefly vs. renilla luciferase activity were calculated. * vs. +, *p*<0.05; * vs. #, *p*<0.05.

The results presented in Fig 1 demonstrated that DHA was more potent than other unsaturated fatty acids such as OA in the inhibition of proinflammatory gene upregulation by LPS or LPS plus PA, but the effect of DHA on CER generation is similar to that of OA (Fig 2), suggesting that a mechanism other than the inhibition of CER generation plays a key role in the inhibition of proinflammatory gene upregulation by LPS or LPS plus PA. To elucidate the underlying mechanism, we tested our hypothesis that DHA inhibits the upregulation of proinflammatory genes by targeting the transcription of proinflammatory genes. We transfected macrophages with plasmids containing 1168-bp IL-6 promoter sequence with the luciferase reporter gene prior to the exposure of cells to LPS, PA or LPS plus PA in the absence or presence of DHA. Luciferase activity assay indicated that LPS and PA had a synergy on IL-6 transcriptional activity, but DHA inhibited the effect of LPS or LPS plus PA on IL-6 transcription (Fig 4A). Since it is known that NFκB plays a key role in the expression of proinflammatory cytokines including IL-6 [32], we further determine the effect of DHA on NFκB transcriptional activity. Results showed that DHA significantly inhibited NFκB transcriptional activity stimulated by LPS or LPS plus PA (Fig 4B). To understand the linkage between CER production and proinflammatory gene expression, we determined if CER stimulates NFκB transcriptional activity. Results showed that C2-CER, a cell membrane permeable CER, exerted a similar stimulation as LPS on NFκB transcriptional activity (Fig 4C).

### CER de novo synthesis is involved in the upregulation of proinflammatory genes by LPS and PA

**Fig 5:**
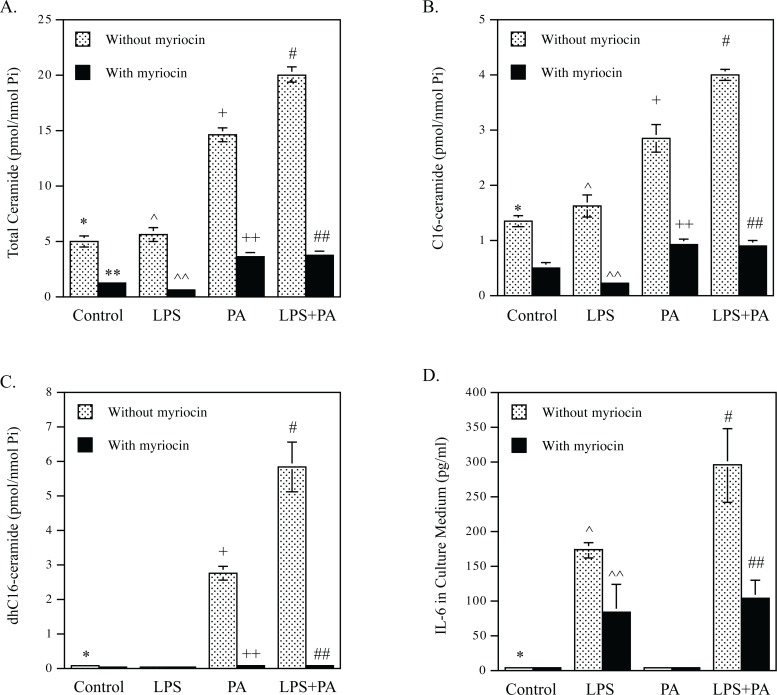
Myriocin inhibits IL-6 secretion stimulated by LPS or LPS plus PA. A-C. RAW264.7 macrophages were treated with 1 ng/ml of LPS, 100 μM of PA or both LPS and PA in the absence or presence of 10 μM of myriocin for 12 h. After the incubation, total (A), C16-CER (B) and dhC16-CER (C) were quantified using lipidomics. D. RAW264.7 macrophages were treated with 1 ng/ml of LPS, 100 μM of PA or both LPS and PA in the absence or presence of 10 μM of myriocin for 24 h. After treatment, IL-6 in culture medium was quantified using ELISA. * vs. +, *p*<0.01; * vs. #, *p*<0.01; ** vs. *, *p*<0.01; ^^ vs. ^, *p*<0.01; ++ vs. +, *p*<0.01; ## vs. #, *p*<0.01.

The above studies showed that DHA inhibited CER de novo synthesis in macrophages. To determine if the inhibition by DHA of CER de novo synthesis contributes to DHA-suppressed proinflammatory gene expression, we employed myriocin, a specific SPT inhibitor [33], to assess the impact of CER synthesis inhibition on proinflammatory gene expression. Since SPT is a rate-limiting enzyme in the CER de novo synthesis pathway [29–31], we expected that the treatment with myriocin would reduce CER de novo synthesis stimulated by LPS and PA. Indeed, lipidomics analysis showed that myriocin potently reduced the baseline level of total CER by 75% and diminished the stimulatory effect of PA and LPS plus PA on CER synthesis by 76% and 84%, respectively (Fig 5A and Table 4). Myriocin also potently inhibited the synthesis of C16-CER (Fig 5B and Table 4). Strikingly, myriocin nearly abolished dhC16-CER stimulated by PA or LPS plus PA (Fig 5C and Table 4). After confirming that myriocin markedly inhibits CER de novo synthesis stimulated by LPS and PA, we determined how myriocin affects upregulation of proinflammatory molecule expression by LPS and PA. Results showed that myriocin inhibited IL-6 secretion stimulated by LPS and LPS plus PA by 52% and 65%, respectively (Fig 5D). In addition to IL-6, data from PCR array showed that myriocin inhibited the stimulatory effect of LPS and PA on proinflammatory genes including MCP-1, CSF3, IL-1α, IL-1β and COX-2 (data not shown).

**Table 4:** The effect of myricin on CER synthesis stimulated by PA and LPS plus PA.

		dhC16-CER	C16-CER	C18-CER	C20-CER	C22-CER	C24-CER	C24:1- CER	Total CER
Without myriocin	Control	0.06±0.006 ^e1^	1.34±0.102 ^e2^	0.06±0.005 ^e3^	0.06±0.001	0.38±0.013	1.64±0.032	1.22±0.070	4.90±0.010 ^e^
	LPS	0.04±0.004	1.62±0.213	0.09±0.006	0.07±0.011	0.39±0.039	1.77±0.205	1.39±0.137	5.52±0.625
	PA	2.75±0.100 ^a1^	3.54±0.254 ^a2^	3.40±0.069 ^a3^	2.24±0.008	1.64±0.015	1.94±0.004	1.35±0.021	14.55±0.308 ^a^
	LPS+PA	5.83±0.721 ^b1^	3.98±0.016 ^b2^	7.48±0.082 ^b3^	4.06±0.026	1.20±0.069	1.59±0.087	1.08±0.028	19.99±0.108 ^b^
With myriocin	Control	0.03±0.005	0.48±0.059	0.04±0.008	0.03±0.003	0.09±0.011	0.15±0.030	0.39±0.023	1.23±0.136
	LPS	0.02±0.002	0.21±0.010	0.02±0.000	0.01±0.001	0.04±0.003	0.13±0.036	0.19±0.017	0.62±0.069
	PA	0.07±0.003 ^c1^	0.91±0.045 ^c2^	0.30±0.001 ^c3^	0.20±0.009	0.39±0.001	0.87±0.018	0.60±0.015	3.55±0.092 ^c^
	LPS+PA	0.08±0.002 ^d1^	0.89±0.023 ^d2^	0.49±0.047 ^d3^	0.29±0.049	0.31±0.012	0.88±0.096	0.58±0.012	3.67±0.172 ^d^

a, *p*<0.01 vs e

b, *p*<0.01 vs a and e

c, *p*<0.01 vs a

d, *p*<0.01 vs b.

a1, *p*<0.01 vs e1

b1, *p*<0.01 vs a1 and e1

c1, *p*<0.01 vs a1

d1, *p*<0.01 vs b1.

a2, *p*<0.01 vs e2

b2, *p*<0.01 vs a2 and e2

c2, *p*<0.01 vs a2

d2, *p*<0.01 vs b2.

a3, *p*<0.01 vs e3

b3, *p*<0.01 vs a3 and e3

c3, *p*<0.01 vs a3

d3, *p*<0.01 vs b3.

The concentration unit of the values in the table: pmole/nmol phosphate.

## Discussion

A number of clinical studies have shown that supplementation with n-3 PUFAs in obese patients reduced proinflammatory cytokines [14, 15]. Increasing reports in recent years have also demonstrated the beneficial effects of n-3 PUFAs on obesity or diabetes [16, 17]. However, our understanding of the mechanisms whereby n-3 PUFAs inhibit inflammation remains incomplete. In the present study, we utilized a cell model that we had established to demonstrate that PA potently amplifies LPS inflammatory signaling in macrophages. We determined if n-3 PUFAs are capable of antagonizing the boosting effect of PA on LPS inflammatory signaling. Since we have shown in this cell model that LPS and PA stimulate gene expression by increasing CER production via both CER de novo synthesis and SM hydrolysis [9], we also determined how DHA affects CER production. The results clearly demonstrated that DHA was much more potent than OA, POA and LA in the inhibition of the boosting effect of PA on LPS signaling and that DHA inhibited the stimulation of CER production by LPS and PA by targeting de novo synthesis, but not SM hydrolysis.

An intriguing finding from this study is that unsaturated fatty acids such as OA, POA and LA have the similar effect as DHA in the inhibition of PA-increased CER de novo synthesis, indicating that the inhibition of PA-increased CER de novo synthesis is not specific for DHA. Given that DHA is more potent than OA, POA and LA in the inhibition of LPS plus PA-stimulated proinflammatory gene expression, it is suggested that a mechanism other than the inhibition of CER de novo synthesis is involved in the robust inhibition by DHA of the gene expression stimulated by LPS and PA.

In our previous study, we have shown that LPS and PA synergistically stimulate signaling activation that ultimately leads to an increase in NFκB transcriptional activity [9]. It has been well established that NFκB as a transcription factor plays a pivotal role in the induction and maintenance of the state of inflammation that underlies metabolic diseases such as obesity and type 2 diabetes [34]. We thus hypothesized that DHA antagonizes the stimulation by LPS and PA of NFκB-dependent transcription. Indeed, the finding from the present study supports our hypothesis as our results showed that DHA potently inhibited the effects of LPS or LPS plus PA on NFκB transcriptional activity. Remarkably, we found that while PA strongly enhanced LPS-induced NFκB activity, DHA inhibited NFκB activity stimulated by LPS and LPS plus PA by 47% and 87%, respectively (Fig 4B). The potential mechanisms by which n-3 PUFAs inhibit LPS-stimulated NFκB activity have been explored previously by Xue et al., who showed that n-3 PUFAs inhibited NFκB activation by stimulating AMP-activated protein kinase (AMPK) pathway [35]. Additionally, Li et al. also reported that n-3 PUFAs inhibited LPS-stimulated NFκB activity by activating peroxisome proliferator-activated receptor gamma [36].

While it is clear that the inhibition of NFκB transcriptional activity plays a key role in the suppression by DHA of proinflammatory gene expression, several lines of evidence from our study and others suggest that the inhibition of CER de novo synthesis also contributes to the powerful inhibition by DHA of proinflammatory gene expression.

First, several studies have demonstrated that PA or LPS increases cellular CER content by stimulating CER synthesis and the increased CER synthesis is responsible for the upregulation of proinflammatory gene expression by PA or LPS [30, 37, 38]. PA is known to augment CER synthesis mainly by its conversion to palmitoyl-CoA, a substrate for SPT to synthesize 3-keto-sphinganine that is a precursor of CER [37, 38]. On the other hand, LPS increases CER synthesis by activating TLR4 signaling that upregulates the expression or activities of the enzymes involved in CER de novo synthesis [37]. To demonstrate linkage between CER synthesis and the proinflammatory gene expression, these studies showed that treatment of cells with pharmacological inhibitors such as SPT inhibitor myriocin or CER synthase inhibitor fumonisin B1 [39] dampens the stimulatory effect of LPS or PA on proinflammatory gene expression. Our present study also showed that myriocin strongly inhibited IL-6 secretion stimulated by LPS or LPS plus PA. Second, our previous study [9] showed that myriocin or fumonisin B1 significantly reduced nuclear content of p65, a major subunit of NFκB [40], indicating that the elevated CER de novo synthesis results in the increased nuclear translocation of NFκB. Moreover, the role of CER in NFκB transcriptional activity is also confirmed by our finding from the present study that C2-CER stimulated NFκB transcriptional activity. Demarchi et al. also demonstrated that C2-CER activated NFκB subunits p50 and p65 in NIH 3T3 cells [41]. However, since C2-CER has a very short fatty acid chain that is quite different from the major intracellular CER such as C16-CER, it may not have all the biological properties of those CERs generated by CER de novo synthesis or SM hydrolysis. Third, our PCR analysis showed that although the combination of LPS and PA is much stronger than LPS alone in stimulation of gene expression, the degree of the inhibition by DHA on LPS plus PA-stimulated gene expression was similar to or even stronger than that on LPS-stimulated gene expression (Table 1). For example, while LPS and LPS plus PA increased IL-6 expression by 39- and 196-fold, respectively, DHA inhibited LPS- and LPS plus PA-stimulated IL-6 expression by 96% and 98%, respectively. Similarly, while LPS and LPS plus PA increased MCP-1 expression by 18- and 48-fold, respectively, DHA inhibited LPS- and LPS plus PA-stimulated MCP-1 expression by 36% and 87%, respectively. Obviously, in addition to targeting the LPS-induced inflammatory signaling, DHA also targets a PA-activated mechanism that contributes to proinflammatory gene expression and the mechanism is likely to be CER de novo synthesis.

It is noteworthy that, in contrary to myriocin that targets the baseline of CER de novo synthesis, DHA antagonizes the PA- or LPS plus PA-stimulated CER de novo synthesis. Our present study showed that myriocin potently reduced the baseline level of the CER content by 75% (Table 4), suggesting that it inhibits the fundamental CER synthesis. By inhibiting the fundamental CER synthesis, myriocin drastically inhibited PA- or LPS-PA-increased CER production by 76% and 84%, respectively. Contrarily, DHA had no effect on the baseline level of CER production, but reduced the increased CER production in response to PA or LPS plus PA (Table 2), suggesting that DHA specifically inhibits the mechanisms by which PA or LPS plus PA increases CER de novo synthesis. In fact, our findings on the inhibition of SPT1 mRNA expression by DHA (Fig 2G) support the above notion. Results showed that DHA did not inhibit the baseline level of SPT1 mRNA expression, but abolished the upregulated SPT1 mRNA expression in response to LPS plus PA.

In our previous study, we reported that PA increased the percentage of CERs with shorter carbon chains such as C16-, C18-, C20-, and C22-CER and decreased the percentage of CERs with longer chains such as C24- and C24:1-CER [9]. Interestingly, the combination of PA and LPS further increased the percentage of CERs with the shorter carbon chains but decreased the percentage of the longer carbon chains, suggesting that the CERs with shorter carbon chains may play an important role in the upregulation of proinflammatory cytokines by LPS and PA. From the current study, it is interesting to find that DHA exerted its stronger inhibition on the CERs with shorter carbon chains than the CER with longer carbon chains. For example, DHA inhibited PA- and PA plus LPS-increased C16-CER by 48% and 50%, respectively, but only inhibited PA- and PA plus LPS-increased C24-CER by 16% and 13%, respectively (Table 2).

While we have focused on the role of CER in the inhibition by DHA of proinflammatory gene expression stimulated by LPS and PA, it is important to understand that DHA also exerts actions that are sphingolipid-independent to inhibit proinflammatory gene expression in macrophages. For example, De Boer et al. have reported that DHA inhibits the secretion of MCP-1 and IL-6 by reducing the degree of M1 polarization of macrophages [42]. Komatsu et al. have also shown that DHA suppresses nitric oxide production in interferon-gamma plus LPS-stimulated macrophage by inhibiting the oxidative stress [43]. Therefore, it appears that the regulation of sphingolipid pathway is one of the multiple mechanisms by which DHA exerts anti-inflammatory effects on macrophages.

**Fig 6:**
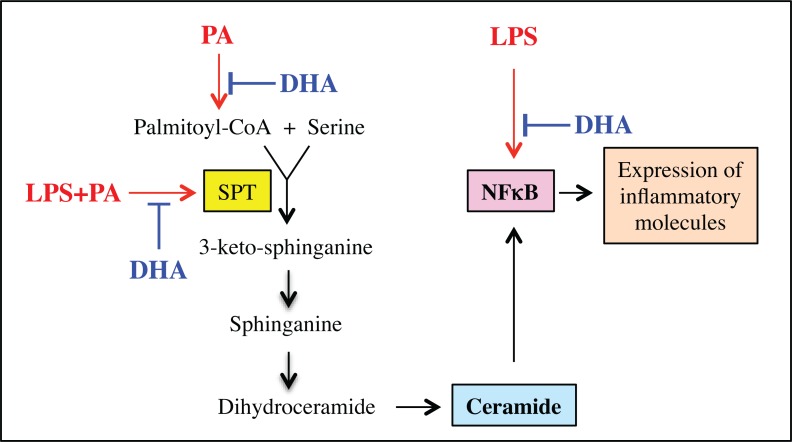
Schematic diagram to show the proposed mechanism involved in the inhibition by DHA of proinflammatory gene expression stimulated by LPS or LPS plus PA in RAW264.7 macrophages.

Taken together, we have demonstrated in this study that DHA antagonizes the boosting effect of PA on LPS inflammatory signaling in macrophages by targeting NFκB-dependent gene transcription and CER de novo synthesis (Fig 6). These findings provide novel insight into the molecular and signaling mechanisms involved in the inhibition of inflammatory response in macrophages by DHA.
